# Targeting ETosis by miR-155 inhibition mitigates mixed granulocytic asthmatic lung inflammation

**DOI:** 10.3389/fimmu.2022.943554

**Published:** 2022-07-26

**Authors:** Ji Young Kim, Patrick Stevens, Manjula Karpurapu, Hyunwook Lee, Joshua A. Englert, Pearlly Yan, Tae Jin Lee, Navjot Pabla, Maciej Pietrzak, Gye Young Park, John W. Christman, Sangwoon Chung

**Affiliations:** ^1^ Division of Pulmonary, Critical Care, and Sleep Medicine, Department of Internal Medicine, Ohio State University Wexner Medical Center, Davis Heart and Lung Research Institute, Columbus, OH, United States; ^2^ Division of Pharmaceutics and Pharmacology, College of Pharmacy and Comprehensive Cancer Center, The Ohio State University, Columbus, OH, United States; ^3^ Comprehensive Cancer Center, Biomedical Informatics Shared Resources, The Ohio State University College of Medicine, Columbus, OH, United States; ^4^ Comprehensive Cancer Center, Division of Hematology, Department of Internal Medicine, The Ohio State University College of Medicine, Columbus, OH, United States; ^5^ Department of Neurosurgery, McGovern Medical School, University of Texas Health Science Center at Houston, Houston, TX, United States; ^6^ Section of Pulmonary, Critical Care, and Sleep Medicine, Department of Medicine, University of Illinois at Chicago, Chicago, IL, United States

**Keywords:** asthmatic inflammation, neutrophilic asthma phenotype, extracellular trap, miR-155, exosome delivery

## Abstract

Asthma is phenotypically heterogeneous with several distinctive pathological mechanistic pathways. Previous studies indicate that neutrophilic asthma has a poor response to standard asthma treatments comprising inhaled corticosteroids. Therefore, it is important to identify critical factors that contribute to increased numbers of neutrophils in asthma patients whose symptoms are poorly controlled by conventional therapy. Leukocytes release chromatin fibers, referred to as extracellular traps (ETs) consisting of double-stranded (ds) DNA, histones, and granule contents. Excessive components of ETs contribute to the pathophysiology of asthma; however, it is unclear how ETs drive asthma phenotypes and whether they could be a potential therapeutic target. We employed a mouse model of severe asthma that recapitulates the intricate immune responses of neutrophilic and eosinophilic airway inflammation identified in patients with severe asthma. We used both a pharmacologic approach using miR-155 inhibitor-laden exosomes and genetic approaches using miR-155 knockout mice. Our data show that ETs are present in the bronchoalveolar lavage fluid of patients with mild asthma subjected to experimental subsegmental bronchoprovocation to an allergen and a severe asthma mouse model, which resembles the complex immune responses identified in severe human asthma. Furthermore, we show that miR-155 contributes to the extracellular release of dsDNA, which exacerbates allergic lung inflammation, and the inhibition of miR-155 results in therapeutic benefit in severe asthma mice. Our findings show that targeting dsDNA release represents an attractive therapeutic target for mitigating neutrophilic asthma phenotype, which is clinically refractory to standard care.

## Introduction

Over the last two decades, asthma incidence in the United States has increased from 5.5% to 7.8% of the general population and is a major health concern for patients and medical professionals (CDC, Most Recent National Asthma Data, 2019). Asthma is the most common chronic inflammatory disease with several characterized endotypes and phenotypes (1–3). Pathophysiologic mechanisms in asthma and treatment strategies are determined by inflammatory phenotypes (4). Unlike the relatively well-defined mechanisms that result in type 2 high eosinophilic asthma inflammation, those leading to non-type 2 high mixed neutrophilic/eosinophilic asthma are poorly understood, and the specific treatments based on molecular pathogenesis are yet to be developed. Although only 10% of asthmatics are categorized as mixed cellular (5), they account for nearly 200,000 hospitalizations and 3,500 deaths per year in the United States (6). Non-type 2 severe asthma is poorly responsive to corticosteroid therapy, compared to the highly steroid-responsive type 2 eosinophilic asthma (7). Recent evidence indicates that asthma exacerbations resulting from excessive activation of the innate immune responses are manifested as airway neutrophilia, although details of the mechanism are not fully elucidated (8–10). Given the frequency, severity, and disability associated with the neutrophilic or mixed cellular asthma phenotypes, it is crucial to identify molecular mechanisms that contribute to mixed cellular asthma that is poorly responsive to conventional therapy.

Although several mediators have been implicated in neutrophil recruitment to the airways (9, 11, 12), these have not proven to be pivotal in the pathogenesis of neutrophilic and/or mixed neutrophilic/eosinophilic asthma, indicating that there are gaps in our knowledge of the immune response in the airways of those with severe asthma. Therefore, it is important to identify critical factors contributing to increased neutrophils in asthmatic airways of these refractory phenotypes. To understand the complex immune responses of severe human asthma, it is imperative to develop a mouse model of severe asthma that displays mixed granulocytic infiltrates in the airways (7). Although no ideal mouse model provides a comprehensive nature of severe human asthma (13), our model is characterized by mixed eosinophilic and neutrophilic inflammation, which is ideal for determining mechanisms and potential therapeutic targets in corticosteroid-refractory severe asthma.

Neutrophils have an important role in innate defense mechanisms. However, in asthma, airway neutrophils have been associated with disease severity and acute exacerbations (14). Elevated sputum eosinophils and neutrophils are associated with the lowest lung function in asthma (15–17). Specifically, the interaction of eosinophils and neutrophils is associated with a greater decline in lung function compared to any single inflammatory pathway in severe asthma (17). Th1/IFN-γ and Th17/IL-17 pathways play critical roles in neutrophil recruitment to the lungs in both neutrophilic and mixed neutrophilic/eosinophilic severe asthma (18–20). The bacterial second messenger cyclic (c)-di-GMP has been shown to be a mucosal adjuvant that induces a Th1–Th17 response (21, 22). Upon activation, neutrophils release chromatin fibers, referred to as extracellular traps (ETs), consisting of host double-stranded (ds)DNA, histones, and granule contents (23). Web-like structure ETs have antimicrobial defense activity by ensnaring microbes in a sticky matrix of extracellular chromatin and bactericidal proteins (24) but also have physiologic and pathologic roles in asthmatic airways (25–27). Although multiple lines of evidence showed that ETs released by activated neutrophils and eosinophils increase asthma severity (26, 28, 29), the mechanisms by which ETs influence the progression of mixed neutrophilic/eosinophilic severe asthma have not yet been defined.

Our study shows that ETs were released in the bronchoalveolar lavage fluid (BALF) of patients with asthma who underwent subsegmental bronchoprovocation with allergen (SBP-AG) challenge. In a murine asthma model that resembles the complex immune responses identified in severe human asthma, we observed that microRNA (miR)-155 regulates the release of ETs, which is associated with the severity of allergic immune responses. Using a serum-derived exosome-based oligonucleotide delivery system, we blocked the release of ETs *in vivo* and attenuated the severity of allergic lung inflammation in the mouse model. Our results provide strong pre-clinical evidence that targeting host dsDNA release is an effective therapeutic approach for mitigating mixed neutrophilic/eosinophilic asthma lung inflammation and alleviating severe airway hyperresponsiveness.

## Materials and methods

Detailed methods are described in the **Supplementary Material**.

### Subsegmental bronchoprovocation with allergen bronchoscopy protocol

This protocol was approved by the Institutional Review Board (IRB) of the University of Illinois (Chicago, IL, USA), and an investigational new drug (IND) was obtained from the Food and Drug Administration (FDA) for bronchoscopic administration of allergens to volunteers. The details of the protocol were described in our previous publication (30, 31). In brief, we recruited study subjects who are on step 1 asthma therapy according to the National Asthma Education and Prevention Program (NAEPP) Asthma Guidelines. After informed consent was obtained, subjects underwent screening for inclusion and exclusion criteria, which included skin prick testing for dust mite, short ragweed, and cockroach allergens. Once the screening was complete, BAL was performed per standard research guidelines approved by the IRB. Pre-challenge BAL samples were obtained right before SBP-AG. At 48 h after the SBP-AG, post-challenge BAL samples were obtained from the allergen exposed, at the adjacent and contralateral subsegment. The maximum challenge dose for SBP-AG was 5 ml of 100 binding antibody units (BAU)/ml or 1:2,000 wt/vol concentration of allergen. The mean age of the UIC was 24 (range 18–36), and the male-to-female ratios were 2:4.

### Mice

C57BL/6, miR-155KO (007745), and PAD4KO mice (030315) were purchased from the Jackson Laboratories (Bar Harbor, ME, USA). Myeloid miR-155 knockout mice and miR-155*^fl/fl^* mice (026700) were crossed with LysM-cre mice (004781, Jackson Laboratories, Bar Harbor, ME, USA) to homozygosity (miR-155*^fl/fl^* LysMcre). Littermates were used as controls. DNA extraction and genotyping were performed as described previously (32). Unless otherwise stated, mice at age-matched 8 to 12 weeks were used in this study.

### Induction of murine asthma model

We used the published triple-allergen (DRA)-induced mouse asthma model with minor modifications (30, 33, 34). DRA mixture includes extracts of dust mite (*Dermatophagoides farina*, 830 µg/ml), ragweed (*Ambrosia artemisiifolia*, 8.34 mg/ml), and *Aspergillus fumigatus* (830 µg/ml; Stallergenes Greer, Lenoir, NC, USA). Mice were sensitized with the DRA allergen mixture on days 0 and 5 by intranasal (i.n.) injection. Seven days later (day 12), mice were challenged with DRA mixture (200 µg) at the same concentration used for sensitization in 20 µl of saline once daily for 3 days/week for 4 weeks. Twenty-four hours following the final challenge, mice were sacrificed, and BALF and lung tissues were collected for further analysis. All sensitized animals had elevated levels of total IgE.

**Figure 1:**
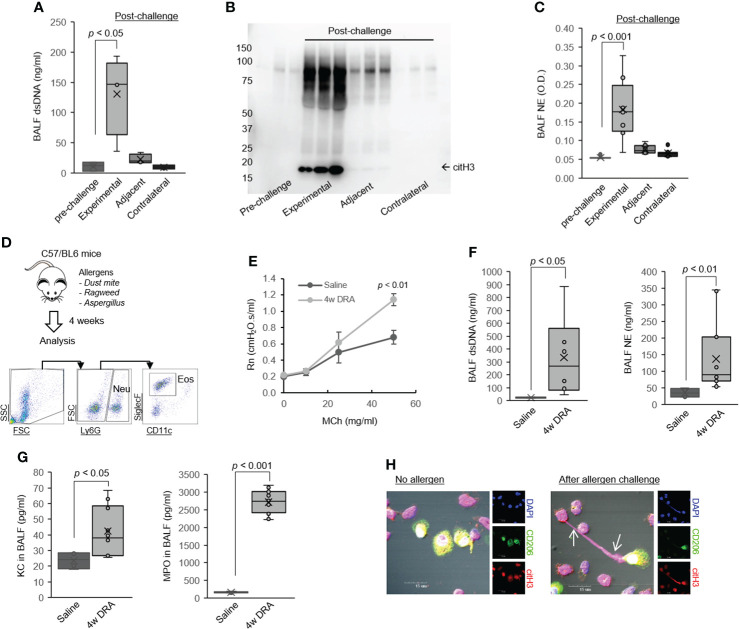
Leukocytes released ET-derived host dsDNA in asthma. **(A)** PicoGreen assays of extracellular dsDNA in the BALF samples obtained pre- and post-allergen challenge in the subsegmental bronchoprovocation with allergen (SBP-AG) protocol (N = 3). **(B)** Western immunoblot for citH3 in BALF samples obtained pre- and post–allergen challenge in the SBP-AG protocol. **(C)** NE from BALF in the SBP-AG protocol. **(D)** Mice were sensitized and challenged by daily intranasal (i.n.) administration of DRA or saline, 3 days a week for 4 weeks. Cell staining for markers of neutrophils (CD45^+^Ly6G^+^) and eosinophils (CD45^+^Ly6G^−^SiglecF^+^CD11c^−^). **(E)** Airway hyperresponsiveness (AHR) in mice subjected to the 4 weeks triple allergen asthma model. Shown are central airway resistance (Newtonian resistance, Rn) values. **(F)** Concentration of extracellular dsDNA and NE in the BALF of mice subjected to triple allergen asthma model (N = 6). **(G)** The level of neutrophil chemokine KC and MPO in BALF from DRA-induced asthmatic mice (N = 6). **(H)** Representative confocal microscopy images of lung macrophages from allergen-exposed mice producing ETs with co-expression of citH3 (arrow). Blue, staining for DNA; red, citH3; green, macrophages. Graphs were plotted as mean ± SE. p-Values were obtained using a two-tailed Student’s t-test. BALF, bronchoalveolar lavage fluid; ET, extracellular trap; NE, neutrophil elastase; MPO, myeloperoxidase.

In the severe asthma model, mice were sensitized with DRA (100 µg) and cyclic-di-GMP (c-di-GMP) (5 µg; Tocris, Minneapolis, MN, USA) intranasally on days 0 and 5. Seven days later (day 12), mice were subjected to three challenge sets involving three consecutive challenges with DRA (100 µg) and c-di-GMP (0.5 µg) with a rest of 4 days in between challenge sets (7). Mice were then euthanized, and features of allergic airway inflammation were assessed, 24 h following the final challenge. The schematic of this model is shown in **Figure 1A**.

### Delivery of small RNA-loaded exosomes *in vivo*

Serum exosomes were isolated according to the manufacturer’s protocol (Thermo Fisher Scientific, Waltham, MA, USA). Nanoparticle tracking analysis was performed to determine the size and concentration of serum exosomes (NanoSight NS300). Modified calcium-mediated transfection was used to introduce small RNAs, including the miR-155 inhibitor (catalog no. MSTUD0080, Sigma, St. Louis, MO, USA) and inhibitor control (catalog no. NCSTUD001, Sigma) into serum exosomes (35). To deliver small RNAs into serum exosomes, 100 pmol of small RNA was loaded into 100 µg of serum EXOs quantified by protein content (36). For *in vivo* experiments, small RNA-loaded exosomes in 30 µl of saline were i.n. instilled into the mouse lung 30 min before the challenge at 3 weeks. One day after the last instillation, mice were sacrificed, and the cellular profile in the BALF was evaluated.

### Measurements of airway hyperresponsiveness

Mechanical properties of the mouse lung were assessed in mice anesthetized with ketamine/xylazine using the forced oscillation technique as previously described (34). Anesthetized mice were mechanically ventilated on a FlexiVent computer-controlled ventilator (SCIREQ, Montreal, QC, Canada) with 8 ml/kg tidal volume at a frequency of 150 breaths/min and 2–3 cmH_2_O positive end-expiratory pressure. Continuous EKG and pulse oximetry monitoring (Starr Life Sciences, Oakmont, PA, USA) was performed. Pressure and flow data (reflective of the airway and tissue dynamics) were used to calculate lung resistance, static lung compliance, and dynamic lung compliance at baseline with the use of the single-compartment and constant phase model. Standardized recruitment maneuvers were performed prior to each physiologic measurement to prevent atelectasis and standardize volume history. Maximal airway responsiveness was measured following exposure to increasing doses of nebulized methacholine (MCh) (0–50 mg/ml).

### DsDNA measurement in bronchoalveolar lavage fluid

DsDNA was measured in the acellular fraction of the BALF, which was obtained after a double centrifugation and supernatant collection. Levels of dsDNA were determined with Quant-iT PicoGreen dsDNA reagent (Invitrogen, Carlsbad, CA, USA) according to the manufacturer’s protocol. For immunoblot analysis for citH3 to detect ETs, anti-citH3 (ab5103, Abcam, Cambridge, UK) was used.

### Single-cell RNA sequencing

Single cells were obtained from asthma lung tissues by mechanical and enzymatic dissociation (33). After enzymatic digestion, the cells were placed in an inactivation buffer (Roswell Park Memorial Institute (RPMI) 1640 + 10% fetal bovine serum (FBS)) and filtered to remove any remaining tissue, the filtrate was centrifuged at 300*g*, the cell pellet was suspended in red blood cell (RBC) lysis buffer and resuspended in inactivation buffer, and viability was determined to be 85%–95% before submitting for approximately 0.75 to 1 × 10^5^ cells for single-cell RNA sequencing (scRNA-seq) analysis. Library preparation was carried out using 10x Genomics Chromium controller (10x Genomics). Chromium 10x Genomics single-cell library was sequenced as 150 base pair single-end reads on an Illumina NovaSeq 6000 (Illumina, Inc., San Diego, CA, USA), with cells from each of the two microchips sequenced across three flow cell lanes.

### Bioinformatics analysis for next-generation sequencing data

Single-cell data in feature-barcode matrices were processed using Cell Ranger 6.0.0 (37) using the GRCm38 (mm10) genome (downloaded March 2021 refdata-gex-mm10-2020-A build from10x) to identify unsupervised cell clusters. The data were natural log transformed and normalized for scaling the sequencing depth to a total of 1e4 molecules per cell, followed by regressing out the number of UMI using Seurat 4.0.4 (38). Briefly, cells were selected based on RNA content thresholds: greater than 300 RNA features, less than 2,000 unique expressed genes (nFeature_RNA), and mitochondrial gene content less than 25% using the “subset” function. Preprocessed data from the six samples were integrated using the Seurat function, the FindIntegrationAnchors function, and IntegrateData using 20 principal components as default parameters normalized with NormalizeData function. The variable features were found with FindVariableFeatures function using 2,000 features. After scaling and principal component analysis (PCA) normalization with default parameters, clustering was performed using Seurat functions FindNeighbors and FindClusters functions using 20 PCs at resolution of 0.8 with the Louvain algorithm. The identified clusters were then visualized using Uniform Manifold Approximation and Projection (UMAP) embedding using 20 PCs. The feature plots were generated using Seurat function FeaturePlot using the RNA assay. Module score was calculated from inputting genes using the Seurat-implemented function AddModuleScore and generated using the FeaturePlot function as described (38). The average expression levels of each cluster at the single-cell level are subtracted by the aggregated expression of random control feature sets (200 by default). For dot plot figures, normalized expression values were generated using NormalizeData function from the RNA assay after splitting clusters by condition. This would create a matrix of natural log-normalized gene expression with a scaling factor of 10,000. The size of the circle represents the number of cells in the cluster when the gene was detected. z-Score dot plots were generated using raw expression data using the scale function in Seurat dot plot function. Pathway enrichment analysis for lymphocyte clusters was performed using Ingenuity Pathway Analysis (IPA) (39). Raw data for IPA analysis was found using Seurat implemented function FindAllMarkers using parameters: only.pos = FALSE, min.pct = 0.2, thresh.use = 0.2. IPA pathway z-score of greater than 2 is considered activated, while a score of less than −2 is considered an inhibited pathway. Canonical Pathway figures were generated using ggplot2 [https://cran.r-project.org/web/packages/ggplot2/citation.html] in R. Functional enrichment analysis by cluster was performed using enrichR [https://maayanlab.cloud/Enrichr/] DEenrichRPlot function using the Gene ontology (GO) 2021 pathways and Kyoto Encyclopedia of Genes and Genomes (KEGG) 2019 mouse databases [https://www.genome.jp/kegg/kegg1.html].

### Statistical analysis

Data were represented as box-and-whisker plots unless otherwise noted. Results were expressed as means ± SE. A comparison between two groups was performed with Student’s *t*-test for unpaired variables. Statistical significance is indicated in figure legends. A p < 0.05 was considered statistically significant.

## Results

### Leukocytes release extracellular trap-derived dsDNA in asthma

**Figure 2:**
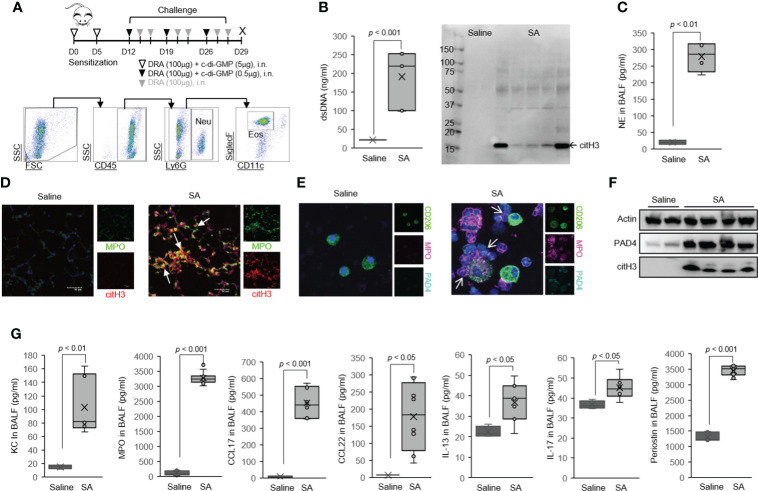
Host dsDNA release was greatly increased in the airways of severe asthma (SA) mice. **(A)** Schematics of the mouse model of SA used in the study. Mice were sensitized and challenged with c-di-GMP and DRA for 3 weeks, and BALF was collected on day 29. Representative gating strategy outlining airway neutrophils and eosinophils in SA mice. **(B)** PicoGreen assay and Western immunoblot for citH3 showing ET-associated DNA present in BALF. **(C)** NE in the BALF of mice in the SA model. **(D)** We observed amorphous, unshaped, extracellular DNA structures that colocalized with citH3 (red) and MPO (green) in severe asthma mouse lungs, as identified by immunofluorescence. **(E)** Representative confocal microscopy images of BAL cells from SA mice producing MPO with co-expression of PAD4 (arrow). Blue, staining for DNA; purple, MPO; green, macrophages; cyan, PAD4. **(F)** Expression of PAD4 as determined by Western immunoblot. **(G)** The level of neutrophil chemokine chemokines and cytokines in BALF from SA mice (N = 6). Graphs were plotted as mean ± SE. p-Values were obtained using a two-tailed Student’s t-test. ET, extracellular trap; BALF, bronchoalveolar lavage fluid; NE, neutrophil elastase.

ETs have been shown to be present in the airways of asthmatics (25, 26). High expression of ETs-derived dsDNA correlates with airway neutrophilia and epithelial cell damage and is related to mucus production (5, 9). Consistent with these findings, our data show that leukocyte-released ET-derived dsDNA presents in BALF of volunteers with asthma who were experimentally challenged with allergens (**Figure 2A**). As illustrated, there was a dramatic increase in extracellular dsDNA in the allergen-challenged site compared with the pre-challenge or contralateral post-challenge sites, with a significantly lower level in the immediate subsegment adjacent to the allergen-challenged site. To confirm that the extracellular dsDNA is derived from ETosis, we measured hyper-citrullinated histone H3 (citH3), a characteristic of ETs. CitH3 was present in the post-challenged sites, the highest at the experimental site (**Figure 2B**). Consistent with this finding, neutrophil elastase (NE) was also higher in the allergen-challenged site (**Figure 2C**).

We also observed an increase in BALF dsDNA and NE levels in a murine model of chronic asthma (**Figure 2F**), in which mice were challenged with the common triple allergen DRA (dust mite, ragweed, and *Aspergillus*) for 4 weeks as our group previously reported (**Figure 2D**) (33). As shown, neutrophils (Neu, CD45^+^Ly6G^+^) and eosinophils (Eos, CD45^+^Ly6G^−^SiglecF^+^CD11c^−^) were detected in the airways of allergen-challenged mice (**Figure 2D**), with was associated with accentuated MCh-induced airway responsiveness (**Figure 2E**). Notably, the levels of neutrophil chemokine CXCL1 (also known as Gro-α in humans and KC in mice) and myeloperoxidase (MPO), a surrogate marker of increased neutrophils, were significantly increased in BALF from the allergen-challenged mice compared to saline-challenged control (**Figure 2G**). Finally, we detected extracellular DNA structures that prominently colocalized with citH3 in pulmonary macrophages and granulocytes from the mouse model of asthma (**Figure 1H**, arrows). These data indicate that recruited leukocytes, neutrophils and eosinophils, are the major cellular source of dsDNA in the asthmatic airways in humans and mice.

### Host dsDNA release is significantly increased in the airways of severe asthma mice

Airway neutrophilia is one of the hallmarks of acute exacerbation or severe subgroups of asthma (14). In order to address this important clinically relevant issue, we employed a mouse model of severe asthma that recapitulates the intricate immune responses of neutrophilic and eosinophilic airway inflammation identified in patients with severe asthma (16, 17, 40). In this severe asthma model (hereafter, SA model), mice are sensitized to the three allergens (DRA) along with c-di-GMP followed by 1 week of rest and then 3 weeks of intranasal challenge (**Figure 1A**) (7). A key feature of this model is increased numbers of eosinophils and neutrophils in BAL samples that were characterized by flow cytometry. In this model, the recruited neutrophils underwent ETosis in the lung, as evidenced by increased BALF dsDNA, citH3, and NE (**Figures 1B**, **C**). Using immunofluorescence microscopy, we observed amorphous and unshaped structures colocalized with citH3 and MPO in severe asthma mouse lungs (**Figure 1D**). Peptidyl arginine deiminase 4 (PAD4) has been shown to be involved in ETosis by hyper-citrullination of histones (5). Notably, high-resolution confocal microscopy indicated the presence of MPO/PAD4-positive neutrophils in the BAL cells of the severe asthma mice (**Figure 1E**), indicating that recruited neutrophils are a cellular source of dsDNA and citH3 in severe asthmatic airways. The level of PAD4 protein was much higher in the whole lung tissue of the severe asthma mice than in the saline-treated control (**Figure 1F**) along with high levels of BAL-KC and BAL-MPO in the severe asthma mice (**Figure 1G**).

Neutrophils and eosinophils were increased in BAL fluids of the mice subjected to the SA model, compared with saline-challenged control (**Supplementary Figures 1A**, **B**), but the SA model had fewer BAL eosinophils compared to the triple allergen (DRA)-only model (**Supplementary Figure 2**). The DRA/c-di-GMP challenge significantly induced the expression of genes associated with Th2 and Th17 immune responses (including *Il13*, *Il17*, *Ifng*, *Kc*, and *Muc5a/c*) compared to the saline-treated control (**Supplementary Figure 1C**). Periodic acid-Schiff (PAS) staining detected that mucus glycol conjugates of goblet cells were also increased in the severe asthma mice (**Supplementary Figure 1D**). Of note, the severe asthma mice had highly increased bronchial hyper-reactivity to MCh (**Supplementary Figure 1E**), compared to that of the triple allergen (DRA)-only model (**Figure 2E**). Total serum IgE and the levels of BALF Th2 cytokines/chemokines such as CCL17, CCL22, IL-13, IL-17, and periostin were elevated in the severe asthma mice compared to the saline-treated control (**Figure 1G**).

### MiR-155 is involved in extracellular trap formation in severe asthma

**Figure 3:**
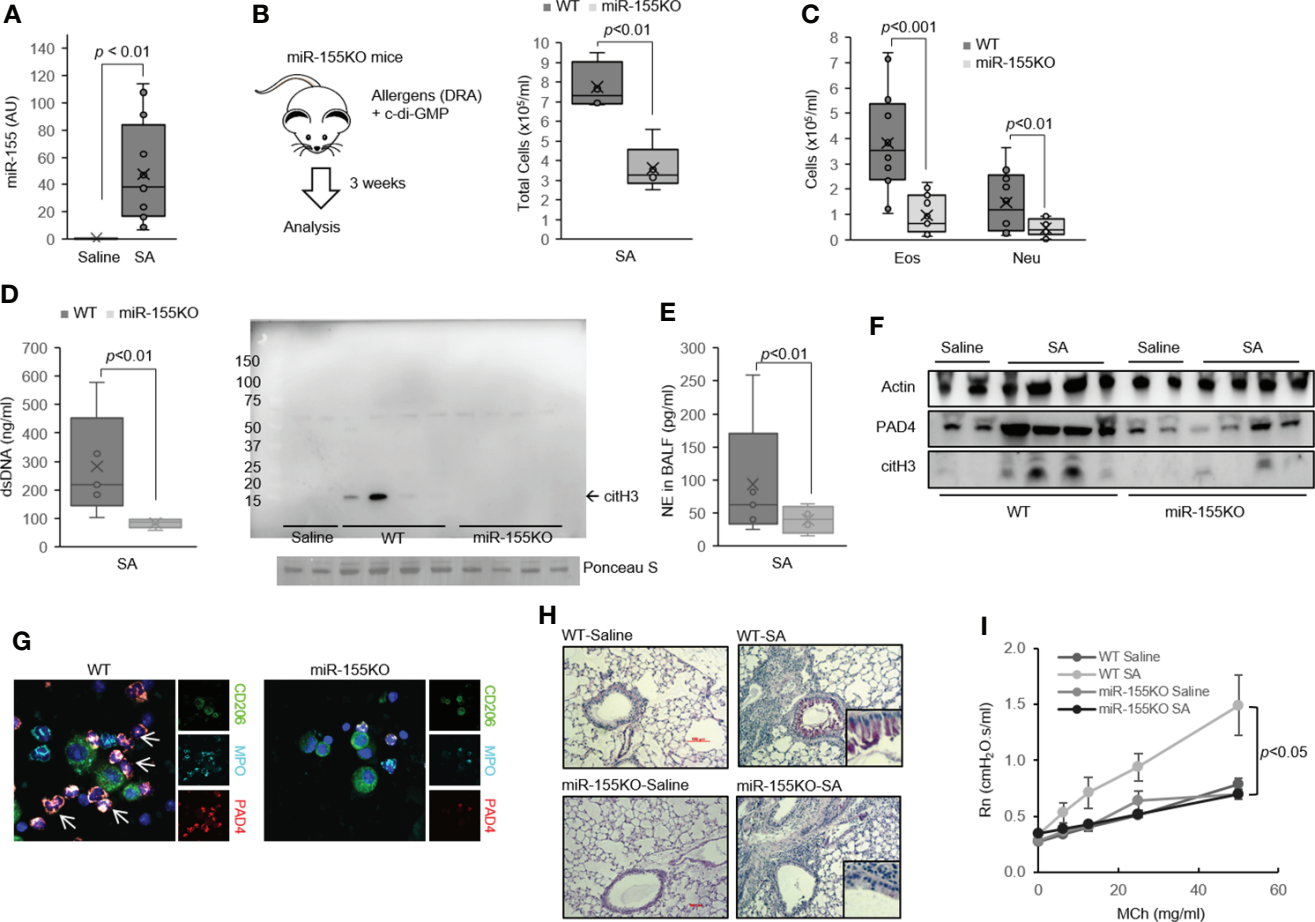
MiR-155 is involved in ET formation in severe asthma (SA). **(A)** Expression of miR-155 in lung from SA mice (N = 3–5). **(B)** Total cells and **(C)** leukocyte differentials were counted in the BALF of the mouse model of SA used in the study (N = 6-8). **(D)** PicoGreen assay and Western immunoblot for citH3 showing ET-associated DNA present in BALF. **(E)** NE in the BALF from WT and miR-155KO mice (N = 5). **(F)** PAD4 and citH3 proteins as determined by Western immunoblotting from SA mouse lung. **(G)** Representative confocal microscopy images of BAL cells from SA mice producing MPO with co-expression of PAD4 (arrow). Blue, staining for DNA; cyan, MPO; green, macrophages; red, PAD4. **(H)** PAS-stained sections from WT (top) and miR-155KO (bottom) mouse lungs subjected to the SA model. Boxed regions are shown enlarged at right. **(I)** Airway hyperresponsiveness (AHR) in WT and miR-155KO mice subjected to the SA model. Graphs were plotted as mean ± SE. p-Values were obtained using a two-tailed Student’s t-test. ET, extracellular trap; BALF, bronchoalveolar lavage fluid; NE, neutrophil elastase; MPO, myeloperoxidase; PAS, periodic acid-Schiff; WT, wild type.

Recent studies indicate that miR-155 plays an indispensable role in Th1/Th2/Th17 immune responses (41, 42). In this study, we hypothesized that miR-155 is involved in ETs formation and related cellular functions in macrophages. We observed that lungs and sorted alveolar macrophages (AMs) from the severe asthma mice contained higher levels of miR-155 than those derived from the saline-treated control (**Figure 3A** and **Supplementary Figure 2B**). Of interest, the myeloid cell-specific miR-155-deficient mice (miR-155^fl/fl^LysM-cre) showed a reduction in extracellular dsDNA level in BALF as compared to control littermates (miR-155^fl/fl^) at a steady state (**Supplementary Figures 3A**–**C**). However, there was no difference in BAL cell numbers (**Supplementary Figure 3D**).

When mice were subjected to the SA model, miR-155 whole-body deficiency (miR-155KO mice) significantly decreased the total number of BAL cells (**Figure 3B**) with a marked reduction in BAL eosinophil and neutrophil counts compared with that in wild-type (WT) mice (**Figure 3C**). Furthermore, the miR-155KO mice had markedly reduced cell-free dsDNA (**Figure 3D**) and NE (**Figure 3E**) in BAL fluids, which are characteristics of ETosis. Consistent with these findings, miR-155KO mouse lungs subjected to the SA model had decreased PAD4 and citH3 expressions (**Figure 3F**). This lower propensity of the miR-155KO mice to ETosis in severe asthma seems to be associated with a decreased number of PAD4-positive neutrophils in BALF (**Figure 3G**). Together, these findings indicate that miR-155 regulates ETosis and is pivotal to neutrophilic lung inflammation such as severe asthma.

The miR-155KO mice subjected to the SA model had significantly decreased mRNA levels of characteristic genes involved in eosinophilic (*Il4*, *Il13*, *Ccl17*, and *Muc5a/c*) and neutrophilic (*Il17*, *Mmp12*, and *Kc*) inflammation compared to the WT counterparts (**Supplementary Figure 4A**). Similarly, the miR-155KO mice subjected to the SA model had decreased PAS staining (**Figure 3H**) and blunted MCh-induced airway responsiveness (**Figure 3I**) as well as marked deficiency in Th2 and Th17 cytokine productions (**Supplementary Figure 4B**). Consistent with these findings, BAL Th2 and Th17 cytokines were also significantly decreased in the miR-155KO mice (**Supplementary Figure 4C**). Collectively, these data indicate that miR-155 regulates Th2 and Th17 allergic lung inflammation *via* ETosis process.

### Inhibition of miR-155 alters eosinophilic and neutrophilic inflammation in the mouse model of severe asthma

Next, we examined whether miR-155 could be targeted to decrease allergic lung inflammation by inducing defective ETosis. Emerging evidence indicates that exosomes can serve as vehicles for therapeutic drug delivery (43, 44). We established a protocol using serum-derived exosomes to deliver the specific inhibitor against miR-155 into the lung *in vivo*, as previously described (35). We purified exosomes from mouse serum and determined the size of serum-derived exosomes and inflammatory lung response *in vivo*. Nanoparticle tracking analysis measurement (NanoSight) showed that the purified serum-derived exosomes were ~78 nm in diameter, as expected from the previous study (45) (**Supplementary Figure 5A**). Incubation of serum-derived exosomes with primary alveolar macrophages showed no cytotoxic effect (**Supplementary Figure 5B**). Serum-derived exosome treatment on mouse lungs did not alter the number of BAL inflammatory cells (**Supplementary Figure 5C**). To evaluate the efficiency of miR-155 inhibition *in vivo*, a synthetic miR-155 inhibitor (mmu-miR-155-5p, Sigma-Aldrich) was loaded into exosomes using modified calcium-mediated transfection and delivered to the lung *via* intranasal instillation (**Supplementary Figure 5D**). As shown, exosome-mediated delivery of the specific miR-155 inhibitor decreased the miR-155 level in the lung, compared with the group treated with a control vector (**Supplementary Figure 5E**).

**Figure 4:**
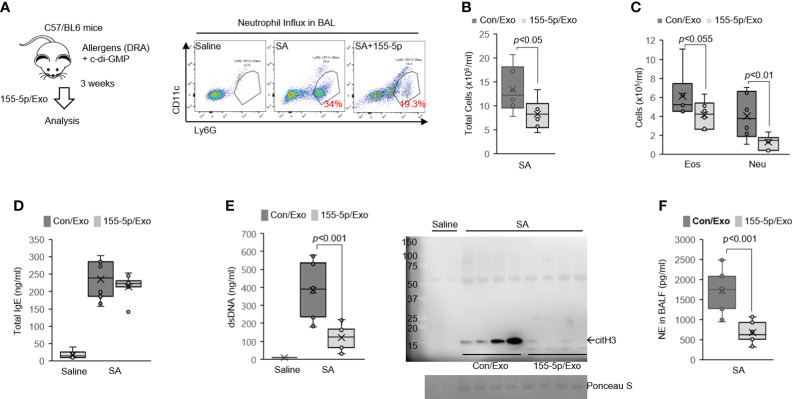
Delivery of miR-155 inhibitor has broad immunosuppressive potential in severe asthma (SA) mice. **(A)** Delivery of miRNA inhibitor *via* serum-derived exosomes into the lung macrophages *in vivo*. Serum exosomes measuring 100 μg transfected with 100 pmol of inhibitor control or miR-155 inhibitor (miR-155-5p) were given. Mice were sensitized and challenged with c-di-GMP and DRA and treated with miR-155 inhibitor or control on days 25, 26, and 27. Neutrophil influx in BAL from SA mice is analyzed by flow cytometry (N = 5-7). **(B)** Total cells and **(C)** leukocyte differentials were counted in the BALF. **(D)** All SA animals had elevated levels of total IgE. **(E)** PicoGreen assay and Western immunoblot for citH3 showing ET-associated DNA present in BALF. **(F)** NE in the BALF of mice to the SA model. Graphs were plotted as mean ± SE. p-Values were obtained using a two-tailed Student’s t-test. BAL, bronchoalveolar lavage; BALF, bronchoalveolar lavage fluid; ET, extracellular trap; NE, neutrophil elastase.

In the SA model, the neutrophil influx into BAL was significantly decreased in miR-155 inhibitor/exosome-treated mice compared with control/exosome-treated mice (**Figure 4A**, 19.3% vs. 34%). The exosome-mediated delivery of miR-155 inhibitor significantly decreased the total number of BAL cells with a marked reduction in BAL eosinophil and neutrophil numbers, compared with control/exosome-treated mice (**Figures 4B**, **C**), although both groups were equally sensitized to the allergens because total serum IgE levels were identical in the SA model (**Figure 4D**). Moreover, BAL-dsDNA and citH3 were markedly decreased in BALF of the miR-155 inhibitor/exosome-treated mice subjected to the SA model as compared with their counterpart control (**Figure 4E**), indicating the critical roles of miR-155 in ETosis. Concordant with the decrease of ETosis, the miR-155 inhibitor/exosome-treated mice had attenuated levels of BAL-NE (**Figure 4F**).

**Figure 5:**
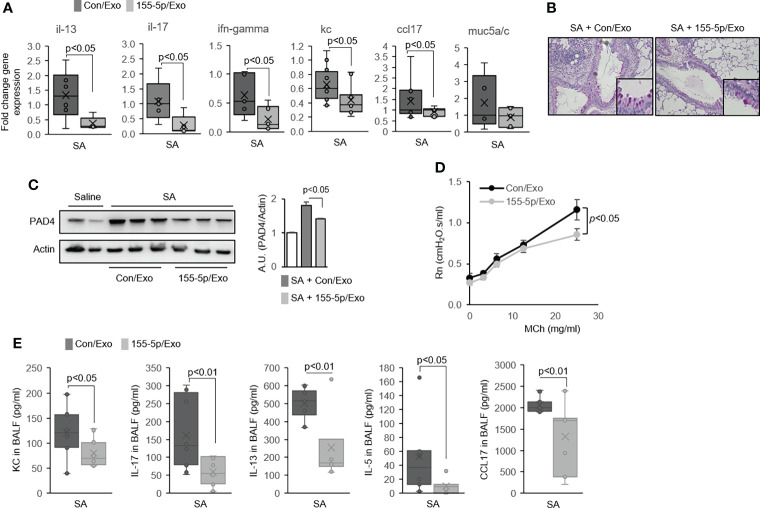
MiR-155 inhibition alters eosinophilic and neutrophilic inflammation in the airways of severe asthma (SA) mice. **(A)** The mRNA expression in lung tissues from Con/Exo- and 155-5p/Exo-treated mouse lungs subjected to the SA model (N = 5–7). **(B)** Representative histologic lung sections stained with PAS. Boxed regions are shown enlarged at right. **(C)** Expression of PAD4 as determined by Western immunoblot. **(D)** Airway hyperresponsiveness (AHR) in Con/Exo- and 155-5p/Exo-treated mice subjected to the SA model. **(E)** The level of cytokines in BALF (N = 5–7). Graphs were plotted as mean ± SE. p-Values were obtained using a two-tailed Student’s t-test. PAS, periodic acid-Schiff.

Next, we examined the asthma features of the SA model with the exosome-mediated delivery of a miR-155 inhibitor. The treatment with a miR-155 inhibitor in the lung in the SA model induced a marked reduction in the expression of *Il13*, *Il17*, *Ifng*, *Kc*, and *Ccl17* genes in the lung (**Figure 5A**); decreased goblet cell hyperplasia (**Figure 5B**); and lowered PAD4 protein expression in the lung as compared to the control group (**Figure 5C**). Airway hyperresponsiveness (AHR) was also markedly lower in the miR-155 inhibitor/exosome-treated mice (**Figure 5D**). Similarly, there was a significant deficiency in Th2 and Th17 cytokine production (**Supplementary Figure 5F**) and the secretion of BAL-cytokines such as KC, IL-17, IL-13, IL-5, and CCL17 in the miR-155 inhibitor-treated group (**Figure 5E**). Together, these data indicate that miR-155 has a pivotal role in promoting ETosis, and miR-155 could be a target to decrease mixed Th1/Th2/Th17 immune responses.

### MiR-155 deficiency alters heterogeneity of lung immune cells in a mouse model of severe asthma

**Figure 6:**
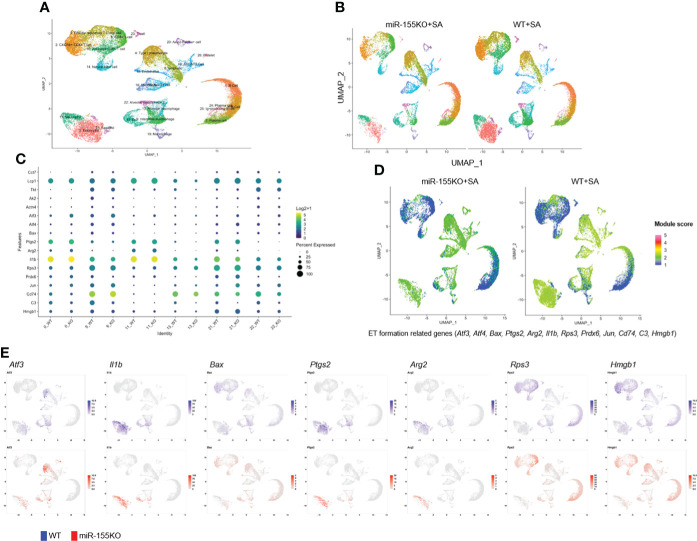
Single-cell RNA-seq analysis of the lung cells in severe asthma (SA) mice. **(A)** Single-cell RNA-seq was performed on single-cell suspensions from whole lungs of mice. Cells were clustered using a graph-based shared nearest-neighbor clustering approach plotted by a UMAP plot. UMAP plot of all scRNA-seq data showing a total of 27 distinct cellular populations that were identified. **(B)** UMAP plots of lung cells from the miR-155KO and WT SA groups (N = 3 per group). All cells were analyzed using canonical correlation analysis with the Seurat R package. **(C)** The expression levels of ET formation-related *Ccl7*, *Lpc1*, *Tkt*, *Ak2*, *Actn4*, *Atf3*, *Atf4*, *Bax*, *Ptgs2*, *Arg2*, *Il1b*, *Rps3*, *Prdx6*, *Jun*, *Cd74*, *C3*, and *Hmgb1* genes are shown. Each dot size corresponds to the percentage of cells in the cluster expressing a gene, and dot color corresponds to the mean expression level for the gene in the cluster. **(D)** UMAP plots compare gene module scores between miR-155KO and WT SA mouse lungs using ET formation-related genes (*Atf3*, *Atf4*, *Bax*, *Ptgs2*, *Arg2*, *Il1b*, *Rps3*, *Prdx6*, *Jun*, *Cd74*, *C3*, and *Hmgb1*). **(E)** UMAP plots show the expression of the indicated ET-related genes in each cluster. UMAP, Uniform Manifold Approximation and Projection; WT, wild type; ET, extracellular trap.

Next, we performed scRNA-seq to investigate the transcriptional profiles of lungs affected by miR-155. Single-cell suspensions of whole lungs were generated from the WT and miR-155KO mice subjected to the SA model (N = 3 each). Consistent with our previous data (**Figure 3C**), the miR-155KO mice had decreased inflammatory cell influx (**Supplementary Figures 6A**–**C**). Cells were immediately encapsulated and barcoded for library preparation using a 10x Genomics microfluidics system, followed by sequencing. We recovered approximately 10,000 individual cells, which were annotated based on established cell markers (38, 46, 47). Unsupervised clustering analysis revealed 27 different clusters corresponding to distinct cell types of the lung, including epithelial, stromal, endothelial, and leukocyte lineages with specific molecular markers (**Figure 6A**). The miR-155KO mice subjected to the SA model showed a decrease in lung immune cells, whereas the numbers of structural cells such as epithelial and endothelial cells were slightly increased compared to their WT counterparts (**Figure 6B** and **Supplementary Figure 6D**). We evaluated the expression of genes associated with asthma-related responses and airway remodeling per cell clusters (10, 47). Several genes were among the most highly expressed factors, including *Egr1*, *Il1b*, *Stat1*, *Rel*, *Tcf4*, and *Nfkb1* (**Supplementary Figure 6E**).

Next, we examined ETs formation-related genes in the identified lung cell clusters. By using dot plots, we showed that the transcripts of ET formation-related genes were substantially downregulated in the miR-155KO mice subjected to the SA model (**Supplementary Figure 6F**). We evaluated the expression of genes associated with ETosis (48–52) including *Atf3*, *Atf4*, *Bax*, *Ptgs2*, *Arg2*, *Il1b*, *Rps3*, *Prdx6*, *Jun*, *Cd74*, *C3*, and *Hmgb1* in leukocyte clusters of the SA model (**Figure 6C**). We also detected low-level expression of several genes in the miR-155KO mice subjected to the SA model. Gene module scores for these signature genes were compared between the miR-155KO and WT mice. The miR-155KO mice subjected to the SA model have less enriched gene module scores of these signature genes compared to their counterpart control (**Figure 6D**). Among these genes, *Il1b* and *Ptgs2* were differentially expressed in granulocyte clusters between the WT and miR-155KO mice. *Rps3* was predominantly expressed in T-cell clusters, whereas *Arg2* was expressed in macrophage clusters (**Figure 6E**). Gene set enrichment analysis using IPA presented the lung leukocyte clusters, which showed decreased activation of pathways of RHOGDI (Rho GDP-dissociation inhibitor) signaling, apoptosis signaling, TWEAK (TNF-related weak inducer of apoptosis) signaling, IL-17 signaling, and PAK signaling, which regulate cellular function including stress-related response and allergic airway diseases 10, 18, 41), during severe asthma in the miR-155KO mice. In contrast, PPAR signaling, PPARα/RXRα signaling, and PTEN signaling pathways in miR-155KO leukocyte clusters were higher than those of the WT counterparts (**Supplementary Figure 6G**).

### PAD4 inhibition results in decreased dsDNA with no effect on granulocytes influx in severe asthma

To further address the functional impact of ETosis on asthma, we used PAD4 knockout mice (PAD4KO) that have defective ETosis (53). PAD4KO mice had a markedly lower level of BALF dsDNA, although inflammatory cells infiltrating the lungs were higher than those of their WT counterpart (**Supplementary Figures 7A–C**). Similarly, the specific inhibitor for PAD4, GSK484 (10 mg/kg, i.p., three times), decreased PAD4 activity in the lung and reduced dsDNA release and NE productions in BALF (**Supplementary Figures 7D**, **E**). However, PAD4 inhibition had no change in the number of total cells or granulocyte counts in the lung (**Supplementary Figures 7F**, **G**) and did not alter miR-155 expression in the SA model (**Supplementary Figure 7H**). These data indicate that PAD4 is downstream of miR-155 with a limited function in dsDNA releases, and miR-155 has a broader impact on asthma phenotypes along with regulating ETosis.

### Intranasal instillation of deoxyribonuclease reduces airway eosinophils but has no effect on airway inflammation in a mouse model of severe asthma

We also tested the potential impact of naked dsDNA on asthma phenotypes with deoxyribonuclease (DNase) I treatment (200 U, i.n., three times) (**Supplementary Figure 8A**). DNase I treatment modestly decreased BAL eosinophils in the SA model, whereas BAL neutrophils remained unchanged (**Supplementary Figures 8A**, **B**). DNase I had a similar partial decrease in airway mucus cell metaplasia and decreased IL-13 and muc5a/c in the lung, suggesting that DNase I did not affect neutrophil response to allergen challenge (**Supplementary Figures 8C**, **D**).

## Discussion

Asthma is a consequence of complex gene–environment interactions, with heterogeneity in clinical presentation (54). Based on the development of relevant biological therapies, type 2-high is the most well-defined endotype of asthma (55). The more severe form of asthma, the mixed granulocytic phenotype, has few endotype-specific therapeutic options, and therefore, further study of its underlying mechanisms is needed. The severe asthma endotype with airway eosinophils and neutrophils is associated with asthma exacerbations and is resistant to inhaled and systemic corticosteroids. To address this important clinically relevant issue, we developed a severe asthma mouse model that recapitulates the immune response that has been identified in the airways of patients with mixed granulocytic asthma. Abundant neutrophils infiltrate the lung, and mixed neutrophilic/eosinophilic inflammation was greatly increased in our severe asthma mouse model, which is consistent with the literature (5, 7, 21, 22). Our data indicate that ETosis is involved directly and indirectly in regulating neutrophil and eosinophil accumulation, which have a pathophysiological role in promoting excessive inflammation and tissue damage. We identified a marked increase in lung eosinophils/neutrophils and extracellular dsDNA in animals exposed to allergens with c-di-GMP. We also discovered that short non-coding miR-155 acts as an early regulator of the release of host dsDNA and ETosis in severe asthma. The severity of the disease is reduced in miR-155-deficient mice compared to their WT counterparts. The neutrophil influx in BAL was decreased in miR-155 inhibitor/exosome-treated severe asthma mice. There was a significantly reduced BALF dsDNA in PAD4 deficient; however, the infiltration of lung immune cells was not decreased. DNase I instillation decreased the intensity of airway inflammation but did not impact miR-155-derived neutrophilia. Together, these findings indicate that miR-155 promotes the release of host dsDNA that contributes to airway damage and mixed granulocytic inflammation.

Outside of the original observation of ETs antimicrobial activity, recent studies have described an assortment of ET-associated immunologic processes and pathologies. ETs contain histones and granular proteins such as NE and MPO. Soluble ET components prime other immune cells to induce sterile inflammation or immune cell recruitment. The released extracellular DNA acts as a danger signal and triggers cytokine production for additional immune response (24). A novel perspective has emerged in light of recent reports, which showed that ETs are generated by both eosinophils and neutrophils in human atopic asthmatic airways (25, 26). Patients with mixed neutrophils and eosinophils in their lungs have more severe airflow obstruction and a higher frequency of exacerbations and are resistant to treatment with corticosteroids (15). Both neutrophils and eosinophils undergo ETosis to promote inflammation and tissue damage in response to many pathogens and pro-inflammatory stimuli. Therefore, key aspects of ETs in the immune response are sufficient to recapitulate many features of asthma pathophysiology. In this study, we focused on the role of ETs in regulating asthmatic airway inflammation directly or indirectly by modulating other immune cell recruitments. Our severe asthma mouse results unequivocally indicate that there is a substantial level of biologically active ETs present in the airways of asthmatics with neutrophil/eosinophil-enriched inflammatory response. It is important to note that increased ETosis associated with mild asthma as well, suggesting that an imbalance of ET formation in the lung may also contribute to enhanced Th2 response in asthma pathogenesis.

MicroRNAs are dynamic post-transcriptional regulators of gene networks. Profiling studies of miRNAs in human biopsy specimens and mouse models of asthma showed differential expression in ~10%–20% of miRNAs (56). MiR-155 is one of the first identified and most studied miRNAs up to date. It is thought to play a role in the functioning of the human immune system (57, 58). MiR-155 is essential for Th2-mediated eosinophilic inflammation through the transcription factor PU.1 (59). Additionally, ILC2 and IL-33 signaling are regulated by miR-155 in Th2 eosinophilic allergic airway inflammation (42). Thus, miR-155 is of considerable interest for understanding immune regulation in allergic asthma conditions. Herein, we showed that host dsDNA release from ETosis in mixed neutrophilic and eosinophilic asthma phenotypes is dependent on miR-155 expression levels. Many neutrophils infiltrated into the lungs, and increased NE productions were significantly reduced in the miR-155KO mice compared to the WT mice. We made the novel observation that the miR-155KO mice were unable to induce both neutrophil and eosinophil influx in the lung in response to severe asthma conditions, which suggests another role for miR-155 in neutrophilic inflammation in severe asthma. MiR-155-deficient mice also showed decreased PAD4 expression levels as compared to the WT mice, which is consistent with the results of *in vitro* study (60). Our data confirm previous *in vitro* data indicating that after stimulation, activation of PAD4 occurs but is regulated by miR-155 in exaggerated ETs generation. These observations predict that miR-155 functions as a positive regulator of ETosis *via* regulation of PAD4 gene expression. We first asked if myeloid-specific miR-155 deficiency is sufficient to recapitulate the phenotype observed in global miR-155 knockout mice, as well as to gauge the effectiveness of inhibitor delivery to rescue the phenotype. However, we show that myeloid-specific deletion of miR-155 in miR-155^fl/fl^LysMCre mice leads to a reduction of BALF dsDNA without affecting macrophage, neutrophil, or eosinophil numbers in a model of severe asthma. This might explain the myeloid lineage in miR-155^fl/fl^LysMCre mice with residual miR-155 in the lungs since in a model of severe asthma, inflammatory cell influx is completely blunted in whole-body miR-155KO mice. Future studies should identify the role of myeloid miR-155 and their associated phenotypes in neutrophilic severe asthma in the lung.

Various clinical trials using miRNA mimetics or anti-miRNAs as therapeutic targets are currently underway and show promising results (61). Our therapeutic approach involving a miR-155 inhibitor has informed the concept to deliver small RNA molecules using serum-derived exosomes into the lung *in vivo*. An exosome-based drug delivery system has recently attracted increasing attention (36). Notably, exosome-mediated drug delivery has the following advantages in comparison with other approaches, such as nanoparticles, liposomes, and viruses. Nanoparticles and liposomes delivered into the lung *via* the i.t. route usually forms aggregate (35). Exosomes are potentially less toxic and less immunogenic compared with exogenous delivery vehicles (62). They also have been shown to be a mode of transport across the blood–brain barrier (63). However, the delivery of exosome-based therapeutics to the lungs *via* i.n. or i.t. remains unexplored. Our results indicate that the exosome-loaded miR-155 inhibitor is successfully delivered *via* i.n. instillation and exerted functions in lungs *in vivo*. Nonetheless, the correct timing of miR-155 inhibition is critical to be functional in the lungs while limiting inflammatory cell influx and tissue damages. A partial targeting miR-155 after severe asthma onset did not produce salutary effects on reducing BALF dsDNA and lung injury.

In the model tested herein, we used DNase I and PAD4 inhibitors to assess the functional importance of ETs in severe allergic asthma. None of these approaches are fully specified for suppression of the heightened Th2/Th17 response. It is important to note that the biological activity of the inhibitors may not be optimal in the lung tissue and could be influenced by factors that are inherent to the experimental models used (64). Treatment with DNase I and PAD4 inhibitor reduced dsDNA release in BALF; however, it had the same global outcome on the development of severe asthma compared to vehicle severe asthma mice, suggesting that ETs could mediate the onset of severe asthmatic airway inflammation in the models tested here.

Single-cell profiling of lung immune cells and identification of molecular pathways in asthma pathogenesis are critical fields of interest in asthma research. This also provides a vital information resource for the immune cellular network in the lung during an asthma exacerbation. Here, our results provide data-driven different transcriptional profiles present in the immune cells of the lung at the single-cell level that contribute to the pathogenesis of asthma and decode the network by using gene set enrichment analysis. In our established severe asthma mouse model, we identified 27 major cell clusters by using SingleR. While numerous immune cells are present in the lungs of severe asthma mice, eosinophils, T lymphocytes, neutrophils, and macrophages are the most abundant cells, whose levels are consistent across different phenotypes of asthma. The profile of the identified cell clusters in miR-155-deficient and WT mice in the presence of c-di-GMP with DRA challenge was not altered, although miR-155KO suppressed c-di-GMP/DRA-induced increased levels of eosinophils and neutrophils. Detailed analysis revealed distinct transcriptional modulation in heightened Th2/Th17 response when compared in mice lacking miR-155. Inflammatory factors and innate/adaptive immune responses are essential in driving the development and exacerbation of asthma. We were able to find the expression of multiple key factors of persistent inflammation, adaptive immune response, and ETosis pathway in our experimental model. These factors critically regulate exaggerated inflammatory responses, inducing asthma exacerbation (10). The pathway analysis supports that miR-155 influences ETosis, at least in part, through significant modulation of neutrophil, eosinophil, and macrophage responses. ET-derived DNA and granule proteins from these myeloid linage cells are key components in driving the pathogenesis of asthma exacerbation in this model. It has become clear that uncontrolled ET formation or their insufficient removal can have serious consequences; however, ET formation pathway is still under investigation.

There are several limitations of this study, which might serve as directions for future studies. Excessive release of a serine protease NE can damage surrounding tissues and contribute to lung dysfunction (65). Neutrophils are the dominant cellular source of NE, but it is also produced by macrophages (66–68). Our group has reported that a majority of the resident cells in the human BALF from our SBP-AG protocol were CD163-positive AMs (30). In the allergen-challenged experimental site, the newly recruited eosinophils appeared to have co-existed with recruited monocyte-derived AMs (but not neutrophils) (69). We reported that the newly recruited macrophage population in the airway is a very different phenotype from these resident macrophages. Remarkably, elevated NE was observed in BALF obtained from human subjects with asthma after SBP-AG protocol without any neutrophil influx. This phenomenon was speculated to occur due to increased innate immune cells (i.e., monocyte-derived AMs) in a mild intermittent allergic asthma model. Further work is required to address the precise role of macrophage-derived NE in allergic lung inflammation. Second, our experiments using mice treated with PAD4 inhibitor and DNase I did not fully reveal miR-155 regulation pathways. Independent evaluation of miR-155 regulation pathways including the PAD4 axis in the global outcome of the development of severe asthma is further needed. Next, although we found that miR-155^fl/fl^LysMCre mice had reduction of BALF dsDNA, the role of myeloid miR-155 and their associated phenotypes in neutrophilic severe asthma in the lung needs to be further studied. Besides, our next study should address how miR-155 modulates cellular function in specific cell subsets in both innate and adaptive immune responses by scRNA-seq in the future.

In summary, ETosis and associated host dsDNA are cardinal features of mixed granulocytic inflammation in severe asthma. Our data indicate that short non-coding miR-155 contributes to the release of extracellular dsDNA, which exacerbates many features of allergic inflammation. We developed a novel therapeutic tool using serum-derived exosomes as oligonucleotide delivery vehicles, leading to the control of the release of ETs *in vivo*. Our single-cell transcriptional analysis of mouse lung immune cells provides an insightful framework for understanding the distinct expression patterns of ET-related genes at the individual cell level. These findings provide pre-clinical evidence that targeting host dsDNA release represents an attractive therapeutic approach for mitigating the inflammatory asthma features for mixed granulocytic severe asthma patients.

## Data availability statement

The datasets presented in this study can be found in online repositories. The name of the repository and accession number can be found below: NCBI Gene Expression Omnibus; GSE203656.

## Ethics statement

The studies involving human participants were reviewed and approved by University of Illinois Center for Clinical and Translational Science. The patients/participants provided their written informed consent to participate in this study.

## Author contributions

JK and SC performed the experiments, analyzed data, and drafted the manuscript. MK, HL, JE, and TL assisted in the mouse and cellular experiments. PS, MP, and PY obtained and analyzed the scRNA-seq data. NP, GP, and JC served as consultants for this project. GP and JC supported the SBP‐AG protocol and assisted with data analyses. SC supervised the project. All authors contributed to the writing of the final manuscript.

## Acknowledgments

This work was supported by grants from the National Institutes of Health (R01HL137224 and R01HL153170), the American Lung Association (RG-416620 and CA-699246), and Internal Medicine at the Ohio State University (Junior Investigator Award). The authors thank the subjects with asthma who volunteered for the SBP-AG protocol and the staff of the Clinical Interface Core at the University of Illinois Center for Clinical and Translational Science (UIC-CCTS) for assistance with patient recruitment, screening, and performing bronchoscopy. In addition, we would like to thank Flow Cytometry Shared Resource, Comparative Pathology and Digital Imaging Shared Resource, Microscopy Resource, Genomics Shared Resource, and Biomedical Informatics Shared Resource (P30CA016058) for their technical assistance.

## Conflict of interest

The authors declare that the research was conducted in the absence of any commercial or financial relationships that could be construed as a potential conflict of interest.

## Publisher’s note

All claims expressed in this article are solely those of the authors and do not necessarily represent those of their affiliated organizations, or those of the publisher, the editors and the reviewers. Any product that may be evaluated in this article, or claim that may be made by its manufacturer, is not guaranteed or endorsed by the publisher.
