# Supplementary material for: Targeting ETosis by miR-155 inhibition mitigates mixed granulocytic asthmatic lung inflammation
Source: Front Immunol. 2022 Jul 26;13:943554. doi: 10.3389/fimmu.2022.943554 (PMC9360579; doi:10.3389/fimmu.2022.943554)
Supplement: Supplementary file 1 [file DataSheet_1.docx]

Supplementary Material

# Supplementary Data

**Supplemental Material & Methods**

**Materials**

Unless otherwise stated, all biochemical reagents used in this study were purchased from Sigma (St. Louis, MO). Antibody against β-actin (#3700) was purchased from Cell Signaling Technology (Danvers, MA). Antibodies against PAD4 (ab214810), citH3 (ab5103), MPO (ab90810) were purchased from Abcam (Cambridge, MA). Antibody against CD-206 (AF2534) was purchased from R&D system (Minneapolis, MN). PE-conjugated anti-SiglecF (552126) was purchased from BD Biosciences (San Diego, CA). APC-conjugated CD11b (101212), FITC-conjugated CD4 (100509), PE-Cy7-conjugated CD11c (117318), BV421-conjugated Ly6G (127628), and PerCP/Cyanine5.5-conjugated CD45 (103132) antibodies were purchased from BioLegend (San Diego, CA). Anti-mouse CD16/CD32 (14-0161-82) was purchased from eBiosciences (San Diego, CA).

**Inhibitors administration**

C57BL/6 mice were given DRA and c-di-GMP 3 days/week i.n. for 3 week. At 3 week, mice were given GSK484 (10 mg/kg, i.p.), deoxyribonuclease I (DNase I, 200U, i.n.), DMSO vehicle, or PBS 3 times along with concurrent DRA and c-di-GMP treatment.

**Flow cytometry**

Cells collected from BALF were incubated with Fc blocking anti-mouse CD16/32 antibody (BD Bioscience) followed by PerCP Cy5.5 conjugated CD45, BV421 conjugated Ly6G, PE-conjugated anti-SiglecF, PE-Cy7-conjugated CD11c, FITC-conjugated anti-CD3, and APC-conjugated anti-CD11b antibodies. Cells were analyzed on a BD LSRFortessa (BD bioscience) where gating was based on respective unstained cell population and isotype matching control antibodies. The data were analyzed with FlowJo software (TreeStar, Ashland, OR).

**Lung histology**

Mouse lung tissue was prepared using pressurized low-melting agarose as descried previously (Chung et al., 2019). Briefly, 1.5% wt/vol low-melting-point agarose was infused through the tracheostomy. The tracheostomy tube was tied and the lung was put into 10% formalin and embedded them in paraffin. Periodic Acid-Schiff (PAS) staining was conducted by the Comparative Pathology and Mouse Phenotyping Shared Resource at the Ohio State University. For immunohistochemical analysis, we deparaffinized and rehydrated lung sections prior to staining. Samples were stained using rabbit (ab64261, Abcam) and mouse (ab64259, Abcam) specific HRP/DAB (ABC) detection kits according to the manufacturer’s protocol. Anti-PAD4 (ab214810, Abcam) and anti-MPO (ab90810, Abcam) were stained at 4 °C overnight.

**Confocal microscopy**

For immunofluorescence staining in vivo, we embedded lungs in optical cutting temperature (O.C.T.) compound (Fisher Scientific) and cryosectioned them. Cytospin slides from BAL sample were used for immunofluorescent staining. Following fixation with 4% PFA, we blocked sections with 2% BSA for 1 hour. We incubated samples with anti-citH3 antibody (ab5103, Abcam), anti-MPO antibody (ab90810, Abcam), anti-PAD4 antibody (ab214810, Abcam), or anti-CD206 antibody (AF2534, R&D system) at 4°C overnight and incubated them with anti-rabbit IgG Alexa Fluor 594 (8889, Cell signaling), anti-mouse IgG Alexa Fluor 647 (4410, Cell signaling), or anti-goat IgG NL493 (NL003, R&D system). Fluorescent images were photographed using Olympus FV 3000 confocal microscope in Campus Microscopy & Imaging Facility at the Ohio State University.

**Measurement of cytokines and total IgE**

The Proteome Profiler™ mouse cytokine array panel A (R&D systems) was used to detect cytokine expression profile in mouse BALF. Cytokines in BALF were analyzed by DuoSet ELISA specific for NE, KC, MPO, IL-5, IL-13, IL-17, Periostin, CCL17, and CCL22 (R&D systems) following the protocols supplied by the manufacture. Serum total IgE was quantitated with Mouse IgE ELISA kit (BioLegend).

**Western blot analysis**

Tissues were lysed in RIPA lysis buffer (Millipore, Temecula, CA) with halt protease and phosphatase inhibitor cocktail (Thermofisher). Lysates containing equal amount of protein were electrophoresed and immunoblotted using appropriate antibodies as described (Chung et al., 2019). Band density was calculated by densitometry analysis (NIH Image J software) and expressed as fold change relative to corresponding loading control, β-actin.

**Quantitative real-time RT-PCR**

RNA was extracted from frozen lung tissues or cell pellets using miRNeasy Mini kit (QIAGEN) or Direct-zol RNA Kits (Zymo Research) according to the manufacture’s instruction. cDNA synthesis with RevertAid First Strand cDNA Synthesis Kit (Thermofisher) and gene expression was measured by the change-in-threshold (ΔΔCt) method based on quantitative real-time PCR in an Roche LightCycler 480 (Roche), normalizing to GAPDH expression as an endogenous control. The following primers were used: GAPDH, 5′-tgcgacttcaacagcaactc-3′ (Fw) and 5′-cttgctcagtgtccttgctg-3′ (Rev); Il-4, 5′-ctggattcatcgataagctg-3′ (Fw) and 5′-tttgcatgatgctctttagg-3′ (Rev); Il-13, 5′-tgaggagctgagcaacatcacaca-3′ (Fw) and 5′-tgcggttacagaggccatgcaata-3′ (Rev); Il-17, 5′-aaggcagcagcagcgatcatcc-3′ (Fw) and 5′-ggaacggttgaggtagtctgag-3′ (Rev); Ifn-gamma, 5′-cggcacagtcattgaaagcct-3′ (Fw) and 5′-gttgctgatggcctgattgtc-3′ (Rev); Kc, 5′-tcgccaatgagctgcgctgtc-3′ (Fw) and 5′-gcttcagggtcaaggcaagcc-3′ (Rev); Muc5a/c, 5′-tacaggctaccagctgtccttgct-3′ (Fw) and 5′-tgcaggtgcaaatggccccac-3′ (Rev); Ccl17, 5′-taccatgaggtcacttcagatgc-3′ (Fw) and 5′-gcactctcggcctacattgg-3′ (Rev); Mmp12, 5′-aatgctgcagccccaaggaat-3′ (Fw) and 5′-ctgggcaactggacaactcaactc-3′ (Rev).

For microRNA quantitative PCR, total RNA was reverse transcribed using miScript II RT kit (QIAGEN) according to instructions. The expression levels of mature miR-155 were analyzed using miScript SYBR Green PCR kit and microRNA-specific RT primers (QIAGEN). The primers used was Mm_miR-155_1 miScript Primer Assay (MS00001701) purchased from QIAGEN. Mature miRNA levels were normalized to RNU6B and quantified using the comparative *Ct* method.

**Study approval**

SBP-AG Bronchoscopy Protocol was approved by the Institutional Review Board of the University of Illinois (Chicago, IL) and an Investigational New Drug (IND) was obtained from the FDA for bronchoscopic administration of allergens to volunteers. All experiments involving mice were conducted with protocols approved by the Institutional Animal Care and Use Committee (IACUC) of the Ohio State University.

# Supplementary Figures and Tables

## Supplementary Figures

**
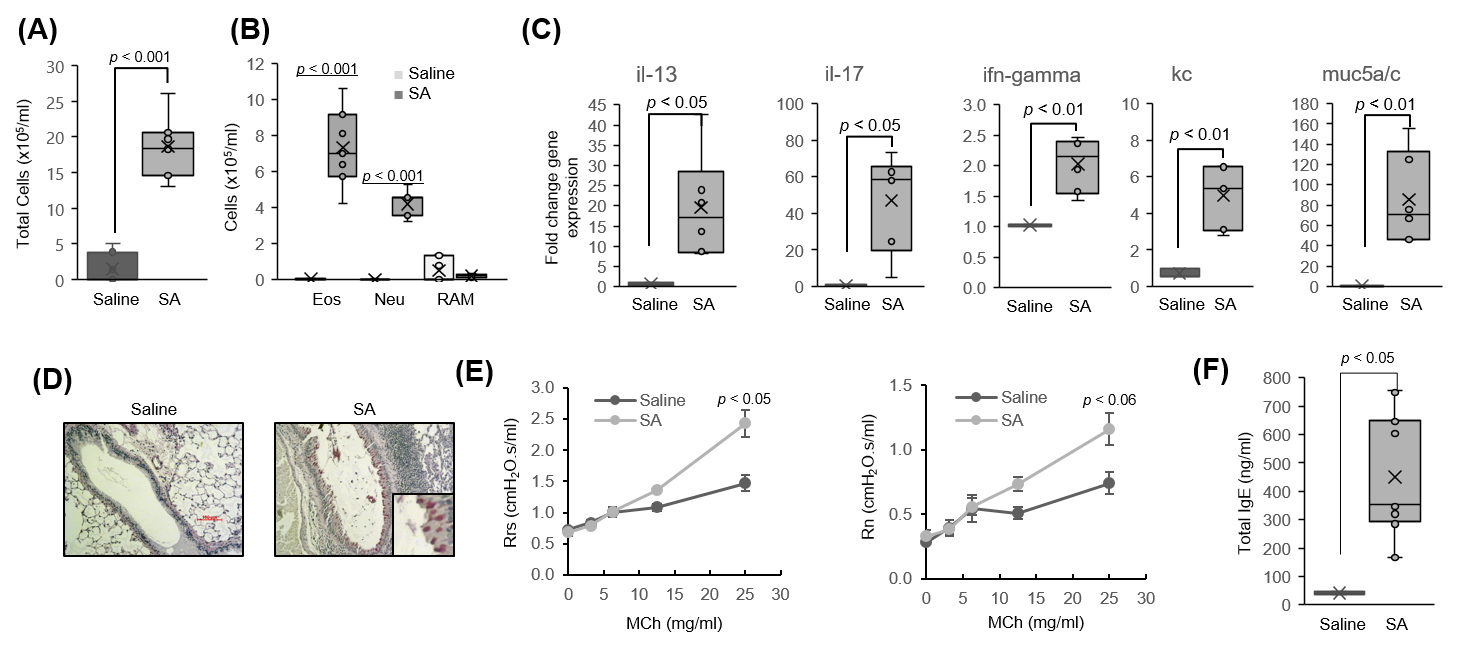
**

**Supplemental Figure 1.** **Eosinophilic and neutrophilic inflammation in the airways of severe asthma (SA) mice. (A)** Total cells and **(B)** leukocyte differentials were counted in the BALF of the mouse model of SA used in the study. **(C)** The mRNA expression in lung tissues from SA mouse model. **(D)** PAS-stained sections from SA mouse lung. Boxed regions are shown enlarged at right. **(E)** Airway hyperresponsiveness (AHR) in mice subjected to the SA mouse. **(F)** All SA animals had elevated levels of total IgE. Graphs were plotted as mean ± SE. p-values were obtained using a two-tailed student t test.


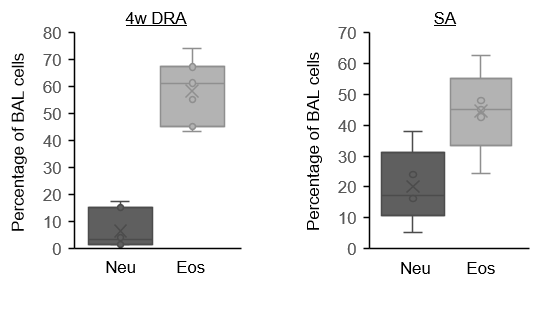


**Supplemental Figure 2. Leukocyte differential counts in the mouse model of asthma.** Percentage of BAL leukocytes were counted in the BALF of the mouse model of DRA-induced asthmatic mice (N=7) and severe asthma mice (N=5) used in the study.


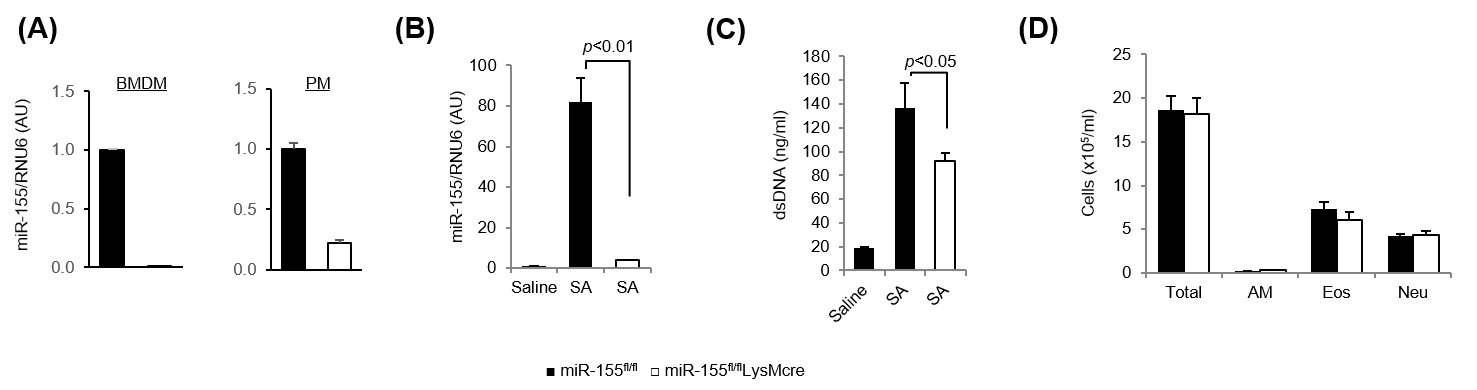


**Supplemental Figure 3. Myeloid miR-155 is not involved in ETs formation in severe asthma. (A)** Loss of miR-155 expression in bone marrow derived macrophages (BMDM) and peritoneal macrophages (PM) from naïve miR-155^fl/fl^LysMcre mice. **(B)** Expression of miR-155 relative to RNU6 in sorted alveolar macrophage (AM, CD45+CD11c+SiglecF+) from miR-155^fl/fl^ and miR-155^fl/fl^LysMcre SA mice. **(C)** Host dsDNA release in BALF from miR-155^fl/fl^ and miR-155^fl/fl^LysMcre SA mice (N=5). **(D)** Total cells and leukocyte differentials were counted in the BALF of the mouse model of SA used in the study (N=7-8). Graphs were plotted as mean ± SE. p-values were obtained using a two-tailed student t test.


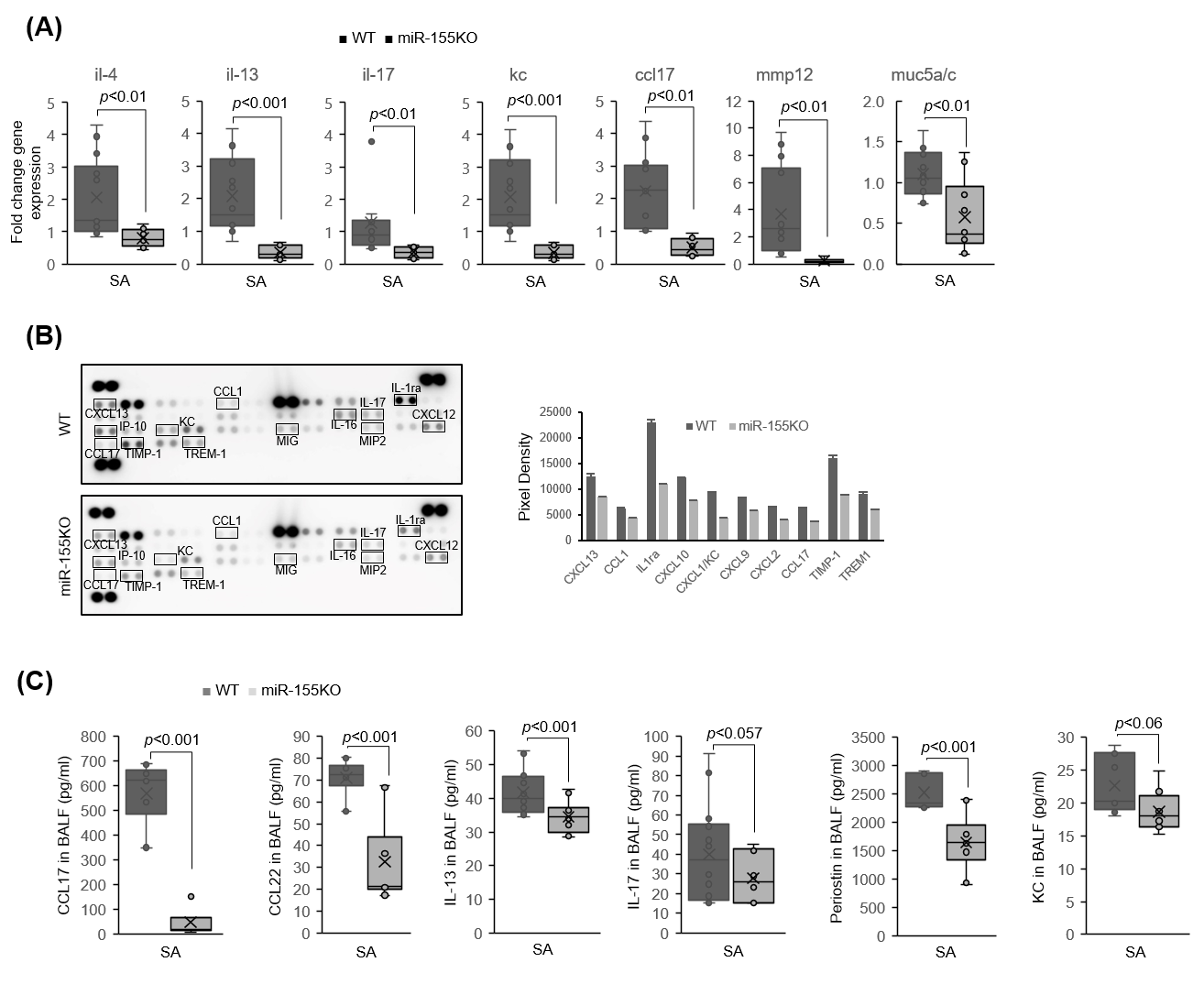


**Supplemental Figure 4.** **MiR-155 is involved in Th2 and Th17 inflammation in the airways of severe asthma (SA) mice. (A)** The mRNA expression in lung tissues from SA mouse model (N=5-7). **(B)** Cytokines were detected in BALF from WT and miR-155KO SA mice was quantified and normalized to WT using an antibody array. **(C)** Cytokine proteins were quantified with ELISA in BALF (N=5-7). Graphs were plotted as mean ± SE. p-values were obtained using a two-tailed student t test.


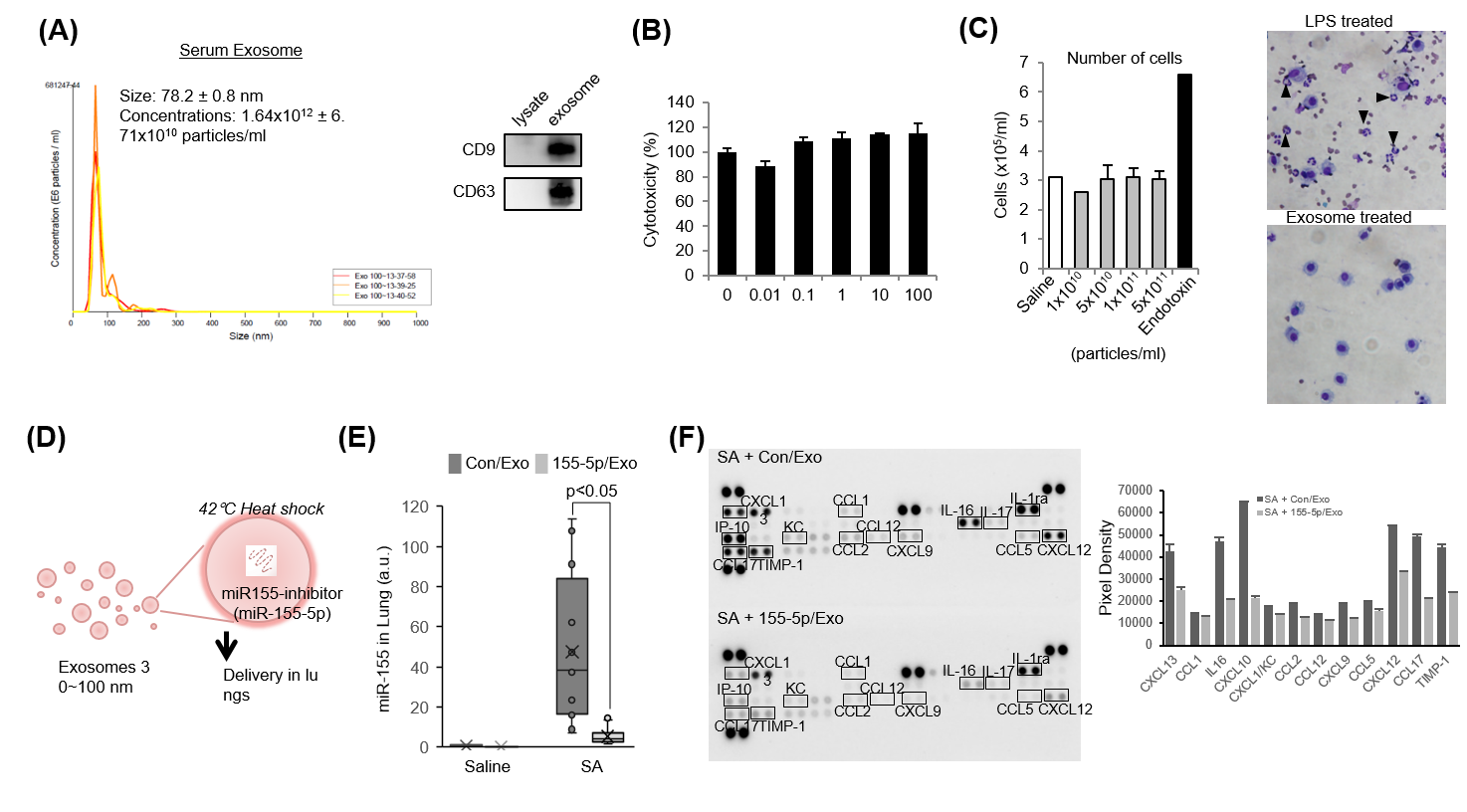


**Supplemental Figure 5. Evaluate exosomes-mediated small RNA molecule delivery system. (A)** The size and concentration profiles of serum exosomes isolated from C57BL/6J mice were measured using NanoSight. Exosome markers (CD9 and CD63) were detected using Western blot. **(B)** Different amounts of serum exosomes were added to murine primary alveolar macrophage (AM, 1x10^6^ cells) and incubated for 24 hr. MTT cytotoxicity assay was measured according to the manufacturer’s instructions. **(C)** Injection of serum exosome has no proinflammatory effects. C57BL/6J mice were given different amounts of serum exosomes or endotoxin (LPS) *via* intranasal injection. One day after treatment, total cells were counted in the BALF (N=3). Representative pictures of BAL cells from LPS- or exosome-treated mice 24 hr after treatment. **(D)** Delivery of miRNA inhibitor *via* serum-derived exosomes (30-100 nm) into the lung *in vivo*. 100 μg serum exosomes transfected with 100 pmol inhibitor control or miR-155 inhibitor (miR-155-5p) will be given. **(E)** The level of miR-155 was detected in lungs from mice were treated miR-155-5p/exosome (N=4). **(F)** Cytokines were detected in BALF from Con/Exo and 155-5p/Exo treated SA mice was quantified and normalized to Con-Exo-treated SA mice using an antibody array. Graphs were plotted as mean ± SE. p-values were obtained using a two-tailed student t test.


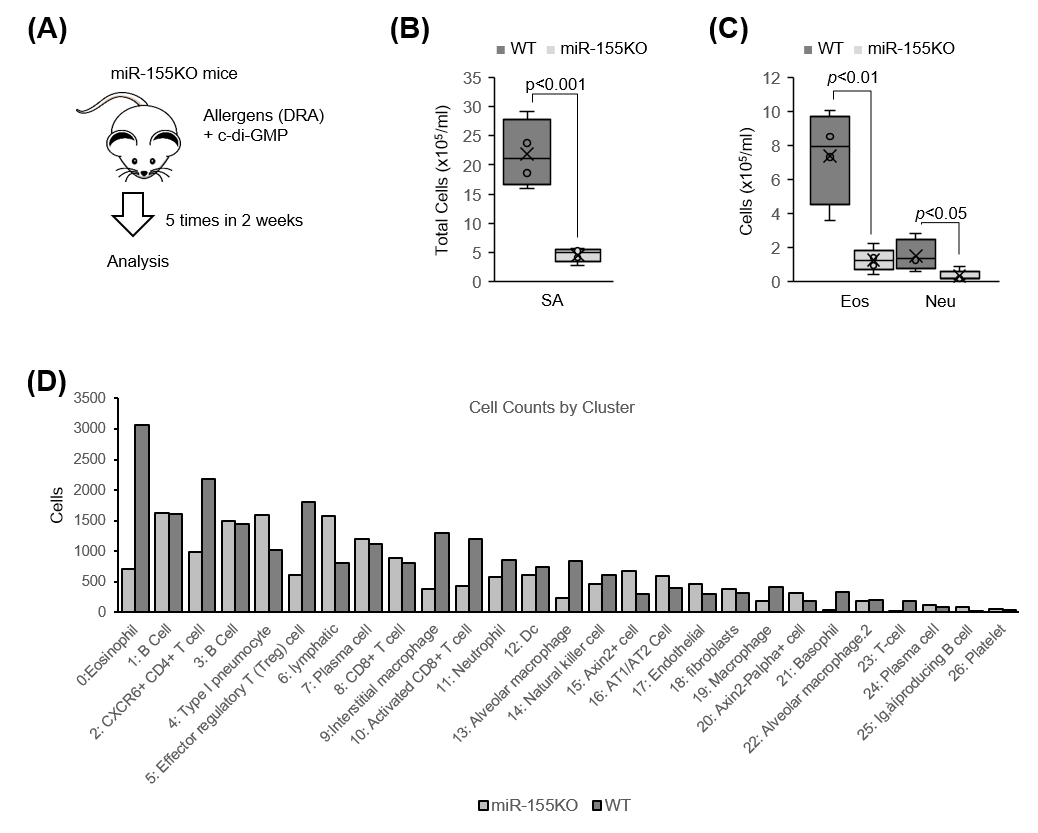


**
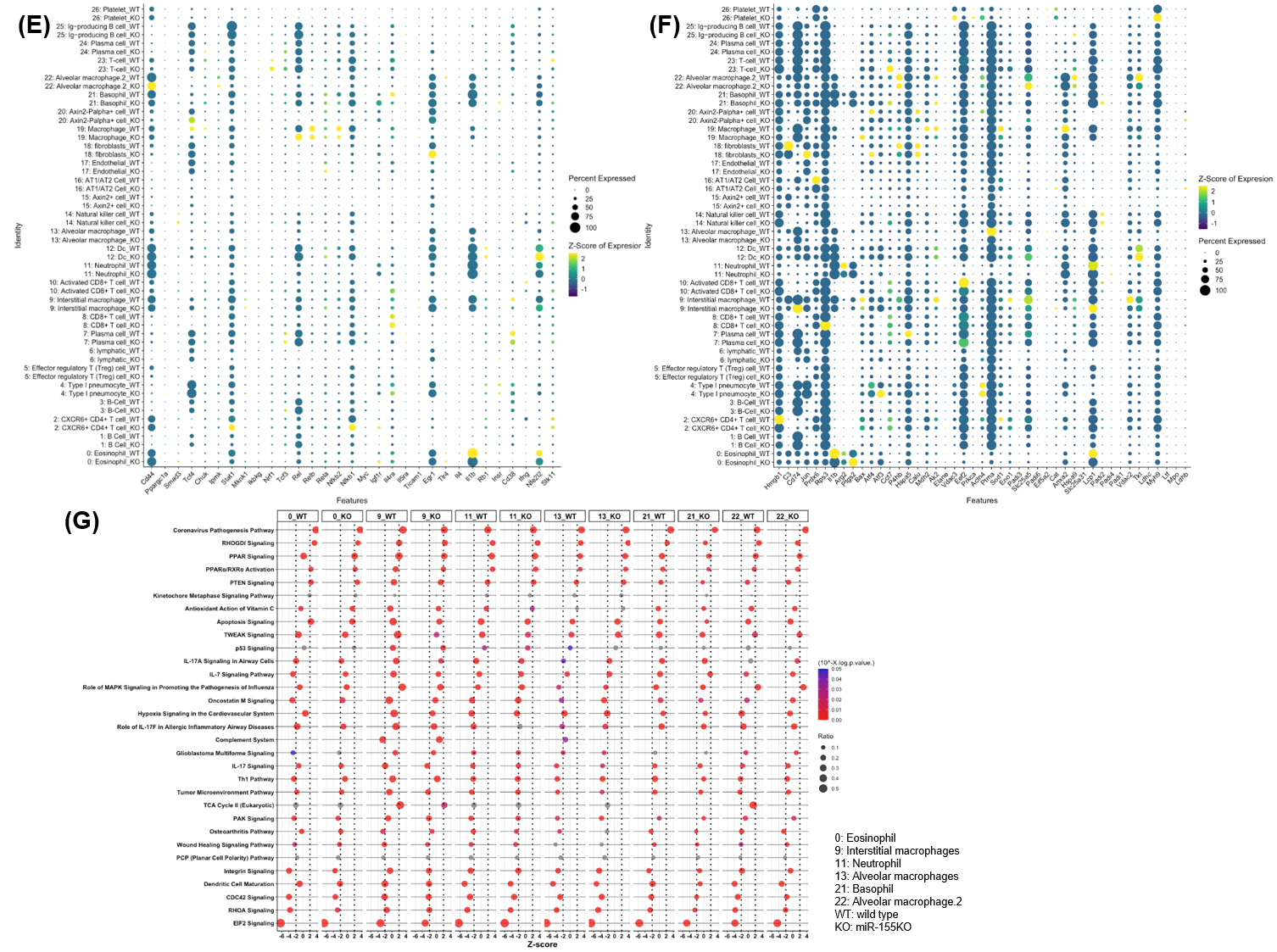
**

**Supplemental Figure 6. Single cell RNA sequencing (scRNA-seq) analysis in severe asthma (SA) mice. (A)** Single cell RNA-seq was performed on single-cell suspensions from the miR-155KO and WT SA groups (N=3 per group). **(B)** Total cells and **(C)** leukocyte differentials were counted in the BALF of the mouse model of SA used in the study. p-values were obtained using a two-tailed student t test. **(D)** Cell levels of scRNA-seq cell cluster in lung. **(E)** Dot plots representing expression levels of asthma-related factors. **(F)** Dot plots representing expression levels of ET formation-related genes from cell clusters in the lung. Graphs are plotted as mean ± SE. **(G)** Predicted canonical pathways in leukocytes clusters (eosinophils, interstitial macrophages, neutrophils, alveolar macrophages, basophils) of WT SA mice compared with respective clusters of miR-155KO mice, generated by Ingenuity Pathway Analysis (IPA) analysis. P values were shown for each pathway and ratio represents the number of genes from the list that maps to the pathway divided by the total number of genes that map to the same pathway.


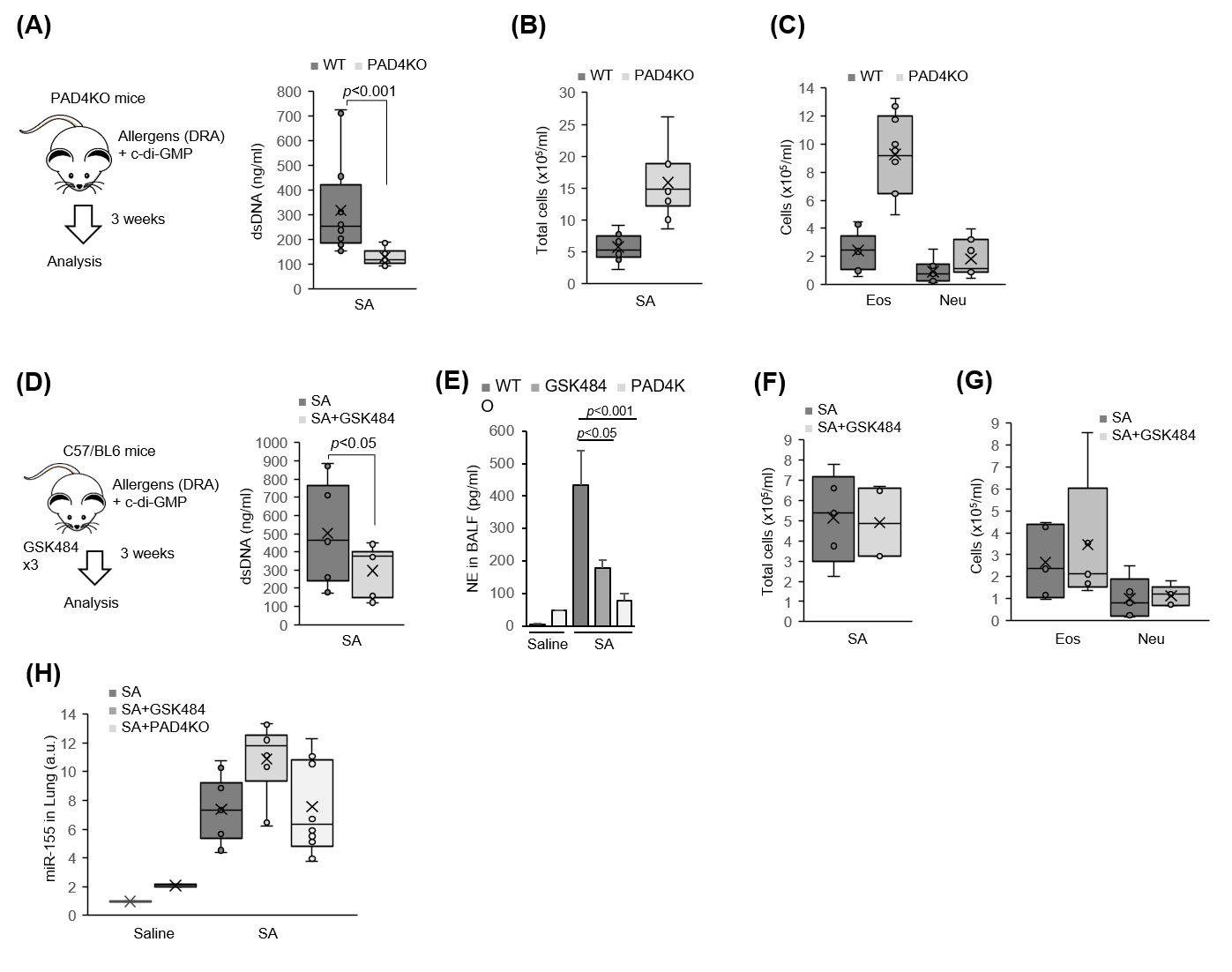


**Supplemental Figure 7.** **PAD4 inhibition alters dsDNA release but not of granulocytes influx in severe asthma (SA) mice. (A)** PicoGreen assays of extracellular dsDNA in the BALF samples obtained from WT and PAD4KO SA mice. **(B)** Total cells and **(C)** leukocyte differentials were counted in the BALF of the mouse model of SA used in the study. **(D)** PicoGreen assays of extracellular dsDNA in the BALF samples obtained from the mouse model of SA used in the study. Mice were sensitized and challenged with c-di-GMP and DRA, and treated with GSK484 or vehicle at day 25, 26, and 27 days. **(E)** The level of NE in BALF from WT, PAD4KO, and GSK484-treated SA mice (N=5-7). **(F)** Total cells and **(G)** leukocyte differentials were counted in the BALF. **(H)** Expression of miR-155 relative to RNU6 in lungs from SA mice used in the study (N=4-7). Graphs were plotted as mean ± SE. p-values were obtained using a two-tailed student t test.

**
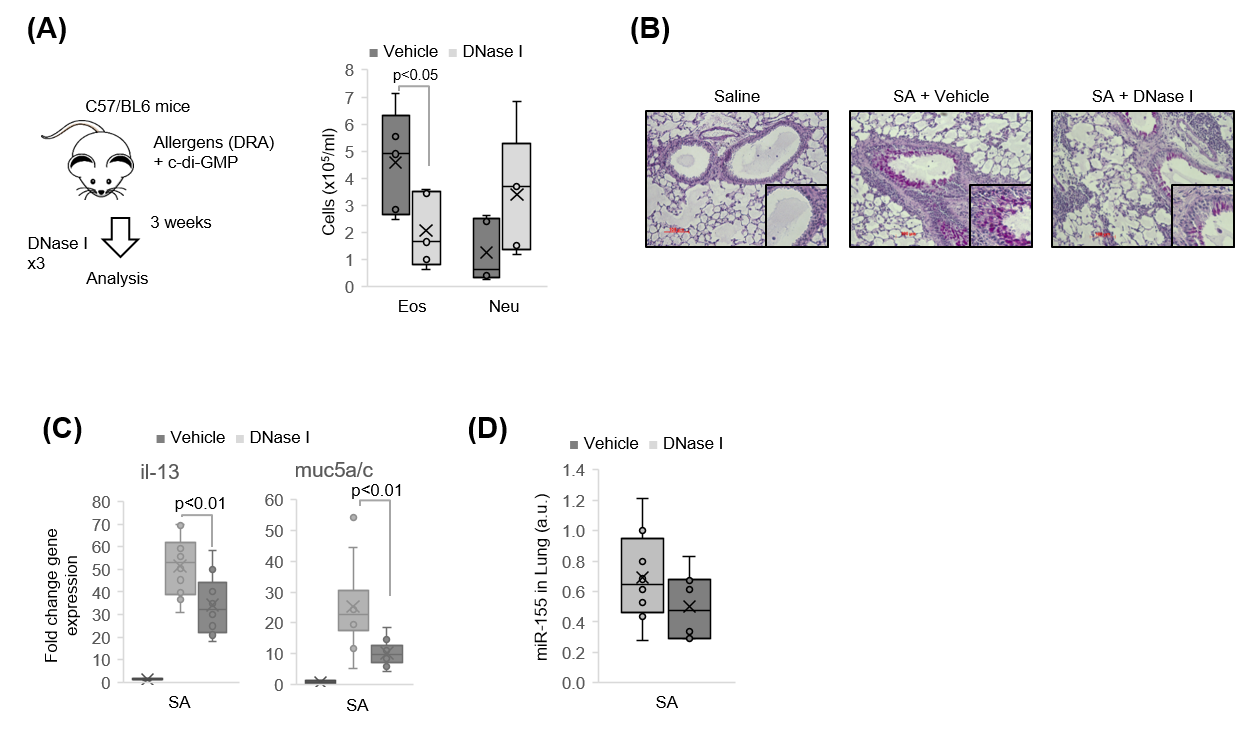
**

**Supplemental Figure 8. Deoxyribonuclease (DNase) I instillation altered levels of airway eosinophils but not of airway inflammation in a mouse model of severe asthma (SA). (A)** Leukocyte differentials were counted in the BALF from DNase I and vehicle treated SA mice (N=4-6). **(B)** PAS-stained sections from SA mouse lung. Boxed regions are shown enlarged at right. **(C)** The Il13 and Muc5a/c mRNA expressions in lung tissues from SA mouse model. **(D)** The level of miR-155 was detected in lungs from mice were treated DNase I. Graphs were plotted as mean ± SE. p-values were obtained using a two-tailed student t test.

**REFERENCES**

CHUNG, S., KIM, J. Y., SONG, M. A., PARK, G. Y., LEE, Y. G., KARPURAPU, M., ENGLERT, J. A., BALLINGER, M. N., PABLA, N., CHUNG, H. Y. & CHRISTMAN, J. W. 2019. FoxO1 is a critical regulator of M2-like macrophage activation in allergic asthma. *Allergy,* 74**,** 535-548.
